# Long-term effect of firing protocols on surface roughness and flexural strength of lithium disilicate glass-ceramic

**DOI:** 10.1590/0103-6440202305496

**Published:** 2023-12-22

**Authors:** Kaíssa da Cunha Lima, Rocio Geng Vivanco, Pedro Roberto Braz Rodrigues, Ana Lúcia Pereira Caetano, Fernanda de Carvalho Panzeri Pires-de-Souza

**Affiliations:** 1 Department of Dental Materials and Prosthesis Ribeirão Preto School of Dentistry - University of Sao Paulo. Av do Café s/n, 14040-904 Ribeirão Preto, SP, Brazil.

**Keywords:** lithium disilicate, thermomechanical cycling, simulated brushing, surface roughness, flexural strength

## Abstract

This study evaluated the effect of different firing protocols on the surface roughness and flexural strength of CAD/CAM lithium disilicate glass-ceramic (LD) after aging methods. Forty-two LD bars of 16 x 4 x 2 mm (IPS e-max CAD, Ivoclar) were randomly separated into two groups according to firing protocols: Single firing-Staining, glazing, and crystallization in a single step; Multiple firings-Crystallization+First staining+Firing+Second staining+Firing+Glazing+Firing. After protocols, initial surface roughness readings were taken (Surfcorder SE1700, Kosakalab). Samples were then randomly separated into three groups (n=7) according to the aging methods they were submitted: Thermomechanical cycling (TMC, ER System, Erios, 1,200,000 cycles, 0.3 MPa, 2 Hz and 5°C/37°C/55°C, 30 s swell time); Simulated toothbrushing (STB, Pepsodent, MAVTEC, 73,000 cycles), and Control (no aging). Final surface roughness readings were done, and samples were submitted to a three-point bending test (OM100, Odeme Dental Research) and fractographic analysis by scanning electron microscopy (EVO-MA10, ZEISS). Data were analyzed (2-way ANOVA, (α=.05). There was no difference (p>.05) in the flexural strength between the firing protocols, regardless of the aging method. STB decreased the flexural strength of samples submitted to multiple firings, different from control (p<.05). Without aging (Control), before TMC, and after STB, LD had lower surface roughness when submitted to multiple firings than to single firing (p<.05). The firing protocols did not affect the flexural strength or the surface roughness of the lithium disilicate glass-ceramic, even after aging. However, toothbrushing negatively affected the flexural strength and smoothed the surface of the ceramic submitted to multiple firings.

## Resumo

Este estudo avaliou o efeito de diferentes protocolos de queima na rugosidade de superfície e resistência à flexão de uma cerâmica vítrea de dissilicato de lítio (DL) para CAD/CAM, após métodos de envelhecimento. Quarenta e dois barras de DL (IPS e-max CAD, Ivoclar) de 16 x 4 x 2 mm foram separadas aleatoriamente em dois grupos de acordo com os protocolos de queima: Single firing-Pigmentação, glazeamento e cristalização em única etapa. Multiple firings-Cristalização+Primeira camada do pigmento+Queima+Segunda camada do pigmento+Queima+Glazeamento+Queima. Após os protocolos, foram realizadas leituras iniciais de rugosidade de superfície (Surfcorder SE1700, Kosakalab). As amostras foram então separadas aleatoriamente em três grupos (n=7) segundo o método de envelhecimento a que foram submetidas: Ciclagem termomecânica (CTM, Sistema ER, Erios, 1.200.000 ciclos, 0.3 MPa, 2 Hz e 5°C/37°C/55°C, 30 s de imersão), Escovação simulada (ES, Pepsodent, MAVTEC, 73.000 ciclos) e Controle (sem envelhecimento). Leituras finais de rugosidade foram realizadas, e as amostras foram submetidas ao ensaio de resistência à flexão em três pontos (OM100, Odeme Dental Research) e à análise fractográfica por microscopia eletrônica de varredura (EVO-MA10, ZEISS). Os dados foram analisados (ANOVA de dois fatores, (α=.05). Não houve diferença (p>.05) na resistência à flexão entre os protocolos de queima, independente do envelhecimento. ES diminuiu a resistência à flexão das amostras submetidas a multiple firings, diferente do controle (p<.05). Sem envelhecimento (Controle), antes da CTM e após ES, LD apresentou menor rugosidade de superfície quando submetido a multiple firings do que a single firing (p<.05). Os protocolos de queima não afetaram a resistência à flexão nem a rugosidade de superfície da cerâmica vítrea de dissilicato de lítio, mesmo após envelhecimento. No entanto, a escovação afetou negativamente a resistência à flexão e poliu a superfície da cerâmica submetida a multiple firings.

## Introduction

Computer-aided design and computer-aided manufacturing (CAD/CAM) systems have become popular over the last years because they offer optimized workflows and high-quality restorations with excellent marginal adaptation, accuracy, durability, and naturality ^1^. These systems frequently use glass-ceramic blocks, highlighting the lithium disilicate glass-ceramic (LD), due to their good mechanical and esthetic properties ^1^. 

Although CAD/CAM technology reduces manufacturing time, additional steps are required for CAD/CAM materials after milling. Monolithic lithium disilicate glass-ceramic requires at least one crystallization firing step to improve its strength and esthetic ^2^. The first firing is essential as it eliminates microcracks and releases tensions associated with finishing and polishing procedures ^3^. Subsequent firings are performed to further enhance the restoration aesthetically through extrinsic characterization ^3^. Stains are applied in multiple layers, with each layer being fired separately. A glaze layer is then applied over the staining layers and fired before cementation to enhance color stability, stain resistance, and overall aesthetic appearance ^4^. After the third firing, the ceramic is ready for cementation, and any subsequent firings are solely to enhance characterization ^3^.

Nonetheless, the current dental practice seeks to simplify protocols to optimize time and costs. As a result, crystallization, staining, and glaze are often performed in a single step through a single firing cycle, not following the manufacturer's recommendations. The number of firing cycles varies significantly among operators, and there is no consensus in the literature regarding the effect of different firing protocols on the performance of LDs ^3^. Dental materials should exhibit high mechanical strength and wear resistance to withstand masticatory forces and parafunction ^5^. The crystallization process causes restructuring and rearrangement of the microstructure of LDs, potentially impacting their physical and mechanical properties ^2^,^6^ ^),^ ^7^ ^,(^ ^8^.

Oral environment conditions may also influence the long-term clinical performance of LDs ^9^. Accelerated artificial aging methods such as thermomechanical cycling (TMC) and simulated toothbrushing (STB) are employed to simulate these conditions and assess their impact on the properties of LDs ^8^ ^),9,10,(^ ^11^.

Considering the above and the lack of studies evaluating the long-term effect of different firing protocols on the flexural strength and surface roughness of lithium disilicate glass-ceramic, the present study evaluated the influence of multiple firings (protocol indicated by the manufacturer) and single firing (protocol frequently used by professionals) on these properties before and after artificial aging by thermomechanical cycling or simulated toothbrushing. The null hypothesis tested was that the firing protocol and aging method would not affect the flexural strength and surface roughness of the lithium disilicate glass-ceramic.

## Material and methods

**Figure 1:**
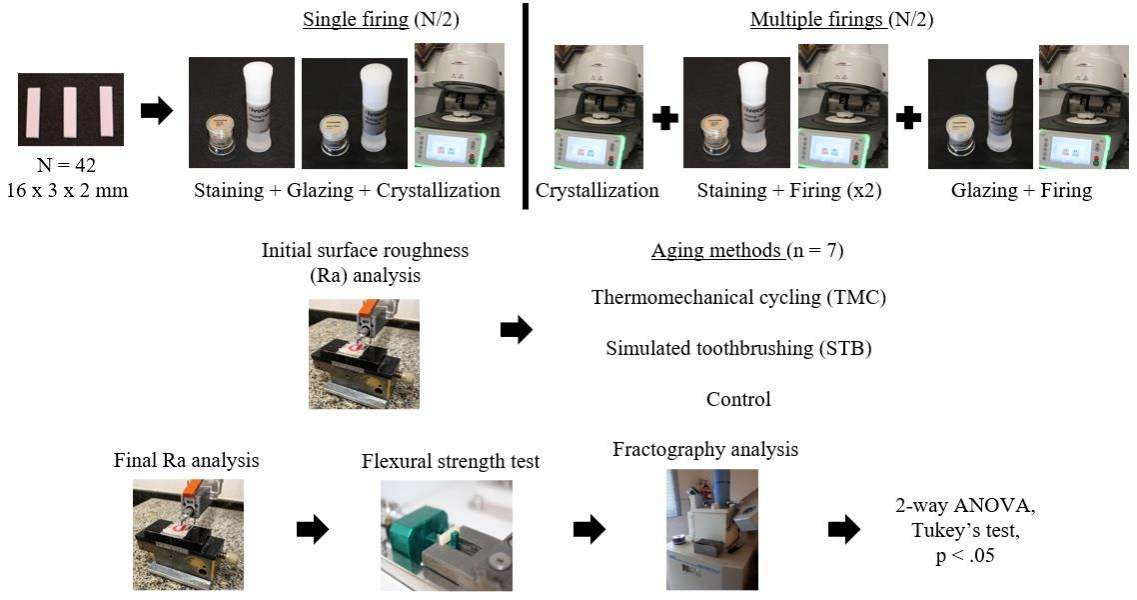
The flowchart presents all the steps of the study.

The sample size (n = 7) was determined from a pilot study, comparing the mean values of flexural strength, with a confidence interval of 95% and power of 80% (www.openepi.com). LD blocks (IPS e-max CAD, Ivoclar) were sectioned into forty-two bars (16 mm x 4 mm x 2 mm) using a diamond disc at low speed, under water-cooling, and in a cutting machine (Isomet 1000, Buehler). The bars were polished manually with SiC sandpaper in decreasing granulation (320, 600, 1200, and 2000 grit) and then, the dimensions were verified using a digital caliper (Absolute Digimatic®, Mitutoyo, Tokyo, Japan). The samples were ultrasonically cleaned (Cristófoli Biossegurança) in distilled water for 5 minutes. The experimental design of the study is shown in Figure 1. Each sample was carefully marked on one surface, while the experiment was conducted on the opposite side. The bars were randomly separated into two groups according to the firing protocol. Staining and glaze layers were applied on the unmarked surface:

Single firing: Staining, glaze, and crystallization in a single step. The stains and the glaze (Ivoclar) were applied with a microbrush and then, the samples were crystallized, following the parameters of the manufacturer (Table 1) (Programat EP5010, Ivoclar)

**Table 1:** Firing protocols

	Stand-by temperature [°C]	Closing time [mm:ss]	Temperature increase [°C/min]	Holding temperature [°C]	Holding time [mm:ss]	Vacuum on [°C]	Vacuum off [°C]	Long-term cooling [°C]	Cool down gradient [°C/mmin]
Single firing									
Single step	403	06:00	90/30	830/850	00:10/07:00	550/830	830/850	710	off
Multiple firings									
Crystallization	403	06:00	90/30	830/850	00:10/07:00	550/830	830/850	710	off
Staining firing	403	06:00	60	770	1:30	450	769	off	off
Glaze firing	403	06:00	60	770	1:30	450	769	off	off

Multiple firings: The samples were crystallized in the first step (without stain and glaze) and then, the first staining layer was applied and fired. After that, a second staining layer was applied over the first one and fired. Finally, a glaze layer was dispensed, and glaze firing was performed. All the firings were done following the parameters of the manufacturer (Table 1) (Programat EP5010, Ivoclar).

### Surface roughness analysis

After the firing protocols, the samples were immersed in distilled water for 24 h. Subsequently, initial surface roughness readings were taken using a rugosimeter (Surftest SJ-201P, Mitutoyo) at 3.2 mm with 3 cut-offs of 0.8 mm, totaling a readout length of 2.4 mm at a speed of 0.25 mm/s. Three readings were done at different locations: one in the center of the sample, and the other 1 mm from the middle on each side.

### Aging methods

After the initial surface roughness readings, the samples were randomly separated into three groups (n = 7) based on the aging method they were subsequently submitted to Control (without aging), thermomechanical cycling, or simulated toothbrushing.

### Thermomechanical cycling

The thermomechanical cycling (ER 37,000, Erios Equipamentos Tecnicos e Cientificos Ltda) was used to simulate changes in temperature and mechanical load applied on the restorations during mastication. The maximum fracture resistance of the LD blocks was calculated, and the equipment was calibrated to apply an axial load 20 % lower than this value. The piston was applied in the middle of the bar and the samples were cycled with a load of 0.3 MPa for 1,200,000 cycles (equivalent to five years of masticatory force) ^12^ at 2 Hz of frequency, with varying temperatures of 5 °C, 37 °C, and 55 °C (± 2 °C) for 30 s dwell time. The parameters were standardized for all the groups.

### Simulated toothbrushing

The toothbrushing was performed using a simulating toothbrushing machine (Pespodent, MAVTEC - Com. Peças, Acess. e Serv. Ltda.). A toothbrush with a soft bristle (Johnson & Johnson Ind. Com. Ltda.) was used for each sample. The ceramic bars were fixed in acrylic resin plates (Acrilpress Ltda) with hot glue to immobilize the samples during the toothbrushing. The toothpaste (Sorriso Dentes Brancos, Colgate-Palmolive) was diluted in distilled water (1:1 ratio) and then, 10 mL of the mixture was dispensed over each sample. The samples were submitted to 73,000 cycles, equivalent to five years of brushing by a healthy individual ^13^, under 200 g load at 365 rpm and 3.8 cm path with back-and-forth movements. Afterward, fragments were washed in running water, dried with absorbent paper, and stored separately.

After subjecting the samples to the aging methods, final surface roughness readings were taken. The initial and final surface roughness values (Ra) were compared.

### Flexural strength test

Following the final surface roughness readings, the dimensions of the samples were measured with a digital caliper and then, they were submitted to a three-point bending test (σ_3-pt_) (OM100, Odeme) at a speed of 0.5 mm/min. The flexural strength was calculated according to ISO 6872 ^14^:

$$\sigma_{f}=3.F.\frac{l}{2}.w.b^{2}$$

Where F is the fracture load (N), l is the span size (15.12 mm), w is the specimen width (mm), and b is the thickness of the specimen (mm).

### Fractography analysis

The fractured samples were observed under scanning electron microscopy (EVO MA10, ZEISS). The samples were fixed in aluminum stubs (Electron Microscopy Sciences), sputter-coated with gold-palladium alloy (Bal-Tec, model SCD 050 sputter coater), and observed at 500x, 1,000x, 2,000x, and 5,000x (20 kV, WD = 30 mm and spot size 28 mm).

### Statistical analysis

Data were tested for normality using Shapiro-Wilk’s test. All the values were considered within a normal distribution, so they were analyzed by Two-way ANOVA (variation factors: aging and number of firings) and Tukey’s test (α =.05).

## Results

The mean comparison of the initial (baseline) and final (after aging) surface roughness among the groups is presented in Table 2. Without aging (Control), LD had lower surface roughness when submitted to multiple firings than to single firing (p = 0.0251). Before TMC, the surface roughness of the samples submitted to single firing was higher than the ones submitted to multiple firings (p = 0.0458). However, after the TMC, the final surface roughness was similar (p > .05) between them. Before the simulated toothbrushing, the firing protocols showed similar surface roughness (p > .05); but after the toothbrushing, the final surface roughness of the samples submitted to a single firing was higher than those submitted to multiple firings (p = 0.0458). There was no difference between the AGING groups, regardless of the number of firings. The interaction between factors was p=0.3830.

**Table 2:** Comparison of the initial and final surface roughness (mean and standard deviation) among the groups (2-way ANOVA, Tukey’s test, p < .05).

Single firing			Multiple firings	
	Initial	Final	Initial	Final
Control	1.07 (0.14) aA		0.71 (0.10) bA	
TMC	1.03 (0.15) aA	1.03 (0.21) aA	0.70 (0.09) bA	0.83 (0.21) aA
STB	0.92 (0.26) aA	1.01 (0.49) aA	0.87 (0.21) aA	0.68 (0.13) bA

Different lowercase letters in the same row indicate a significant difference (p < .05) between the firing protocols within each aging method and time (initial or final). The same uppercase letters in the same column indicate no significant difference (p > .05) between the aging methods within each firing protocol and time (initial or final).

The mean comparison of the flexural strength among the groups is shown in Table 3. There was no difference (p > .05) in the flexural strength between the firing protocols, regardless of the aging method. The aging method did not affect the flexural strength of the samples submitted to single firing (p > .05). Nevertheless, the simulated toothbrushing decreased the flexural strength of the ones submitted to multiple firings, different from the control (p = 0.0445). The interaction between factors was p=0.8895.

**Table 3:** Comparison of the flexural strength (mean and standard deviation) among the groups (2-way ANOVA, Tukey’s test, p <0,05)

	Single firing	Multiple firings
Control	357,65 (33,25) aA	371,76 (75,16) aA
TMC	319,35 (42,03) aA	318,64 (40,04) aAB
STB	303,58 (63,24) aA	299,51 (51,11) aB

Different lowercase letters within the same row and uppercase letters within the same column indicate statistically significant differences (p < .05).

**Figure 2:**
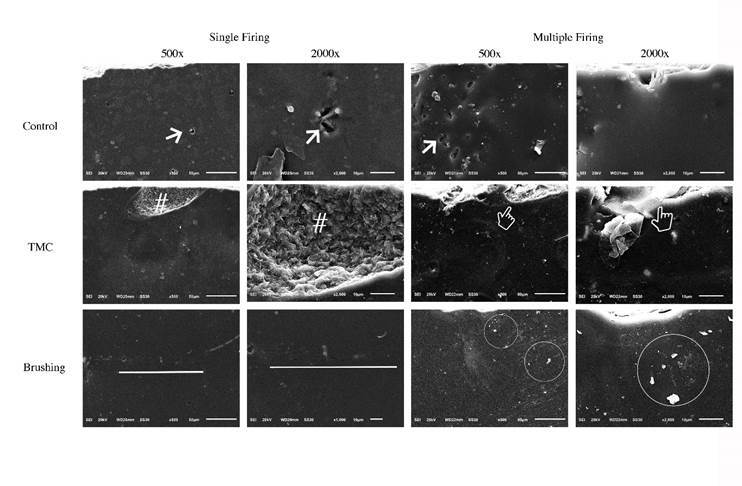
Representative photomicrographs (SEM) of the samples submitted to different firing protocols and aging methods (500× and 2 000×). Narrow = Bubble; Hashtag symbol = Radial crack; Finger point = Chipping; Line = Cracks; Circle = Particles from the toothpaste.

Representative SEM images of the fractured lithium disilicate glass-ceramic after the firing protocols and aging methods are presented in Figure 2. Without artificial aging (Control), LD submitted to a single firing showed fewer and smaller bubbles on the surface than LD submitted to multiple firings. However, both firing protocols produced an irregular surface. After TMC, the samples presented irregularities and deformations on the surface. The glass-ceramic submitted to single firing showed a radial crack probably produced by the loading piston of the equipment. Those submitted to multiple firings failed by irregular chipping also generated by the load point of the TMC, showing greater surface damage. After STB, the LD submitted to single firing presented a greater number of grooves and cracks than the ones submitted to multiple firings, which demonstrated a more uniform surface with some particles, probably from the toothpaste.

## Discussion

The present study evaluated the effect of two firing protocols - A multiple firings protocol following recommended manufacturer instructions and a single firing protocol used by some laboratory technicians without scientific evidence - on the flexural strength and surface roughness of LD submitted to thermomechanical cycling or simulated toothbrushing. The null hypothesis tested was that the firing protocols and artificial aging methods would have no significant influence on the tested properties. Based on the results obtained, the null hypothesis was rejected since the STB decreased the flexural strength of the LD submitted to multiple firings. In addition, in the absence of aging, the LD submitted to multiple firings had lower surface roughness than the one submitted to a single firing. Before the TMC and after STB, samples submitted to single firing presented higher surface roughness than those submitted to multiple firings.

Dental materials that consist of multiple phases, such as LDs with glassy and crystalline phases, require careful processing due to the different reactions of these phases during heat treatments ^15^. During firing cycles, LDs are heated at approximately 850 °C and subsequently cooled to room temperature. These temperature changes can induce residual stress within the ceramics that alters their crystalline structure and decreases their fracture strength ^16^. Although multiple firings introduce more temperature variations that can affect the strength of LDs, the number of firings was not significant in the present study, consistent with previous research findings ^8^,^17^. This can be attributed to the probable absence of any internal structural changes in the material, despite the formation of superficial cracks caused by the heat treatments (Figure 2), as observed in a previous study ^17^.

The present study simulated oral cavity conditions to evaluate the long-term performance of the ceramic and ensure more reliable results. Samples were brushed using a toothbrushing device that simulates the abrasion caused by toothbrush bristles and abrasive particles found in toothpaste during oral hygiene ^10^,^11^ or submitted to thermomechanical cycles that reproduce masticatory load and temperature changes, which can induce tension ^9^. Aging did not have a significant impact on the flexural strength of LD submitted to single firing. However, LD submitted to multiple firings, as recommended by the manufacturer, showed lower flexural strength after STB. A previous study has demonstrated that LD fired once can exhibit higher hardness and fracture toughness than LD fired three or four times ^17^. Thus, LD submitted to single firing would have greater plastic deformation, requiring higher stress to fracture. Conversely, LD submitted to multiple firings would have less tenacity. This, along with the wear caused by the brush bristles and the abrasive particles in the toothpaste, could have decreased its flexural strength ^18^.

LD submitted to multiple firings exhibited lower initial surface roughness than that submitted to a single firing. This can be attributed to the beneficial effects of extended firing, which promote defect healing, decrease flaw depth, and enhance surface smoothness ^19^. However, after TMC, the surface roughness of LD submitted to multiple firings increased. TMC can induce several defects on the ceramic surface, as confirmed by SEM analysis (Figure 2). As a result, the surface roughness values between the two firing protocols became similar ^9^.

Conversely, the brushed LD submitted to multiple firings showed lower surface roughness than the one submitted to a single firing, as corroborated by SEM analysis. The former presented a more uniform surface with fewer cracks after STB (Figure 2). The influence of brushing on the surface roughness of glass-ceramics is controversial. Previous studies have shown that STB can have an abrasive effect on ceramic surfaces ^11^,^20^. Toothbrush bristles and abrasive particles in toothpaste can cause wear on the glaze layer, increasing the surface roughness ^11^,^20^. However, it has also been suggested that glass-ceramics possess high wear resistance, and brushing may primarily target higher peak irregularities, resulting in a smoother surface ^21^.

On the other hand, the adequate application of a glaze layer can prevent the removal of stains and wear in feldspathic ceramics, as glazed ceramics take twice as long to wear compared to unglazed ones ^22^. A similar effect may have occurred in the present study. LD submitted to multiple firings was characterized by two layers of stains and a glaze layer, which may have provided greater protection against brushing compared to the simplified protocol. Therefore, crack-healing promoted by multiple firings, polishing induced by brushing, and the number of stain layers and glaze could explain the surface roughness values obtained after STB in LD submitted to multiple firings.

The present in vitro study simulated oral cavity conditions to which ceramic restorations are exposed; however, it has its limitations. The oral cavity is a complex environment influenced by various factors, including diet and salivary flow, which can alter the oral pH and potentially affect the mechanical properties of LDs ^23^. Therefore, further in vitro and situ studies are necessary to validate the clinical performance of the ceramic.

The results obtained from this study indicate that simpler and faster protocols for daily clinical practice can be an excellent alternative to conventional multi-step protocols. These simplified protocols could reduce both time and resources without compromising the long-term mechanical and physical properties of the LD.

## Conclusion

The firing protocols did not affect the flexural strength and the surface roughness of the lithium disilicate glass-ceramic, after TMC. Without aging, the lithium disilicate glass-ceramic demonstrated a smoother surface when submitted to multiple firings. However, the toothbrushing negatively affected the flexural strength and smoothed the surface of the ceramic submitted to multiple firings.
